# 635. Morbidity and Mortality Associated with Respiratory Syncytial Virus Infections Among the Adult Hospitalized Patients

**DOI:** 10.1093/ofid/ofad500.701

**Published:** 2023-11-27

**Authors:** Ashish Bhargava, Susan M Szpunar, Ambreen Malik, Mamta Sharma, Leonard B Johnson

**Affiliations:** Ascension St. John Hospital; Wayne State University School of Medicine, Grosse Pointe Woods, Michigan; Ascension St. John Hospital, MI; Ascension St John Hospital, Warren, Michigan; Ascension St John Hospital & Medical Center, Grosse Pointe Woods, MI. 48236, Michigan; Ascension St. John Hospital, MI

## Abstract

**Background:**

Respiratory syncytial virus (RSV), commonly a virus of the fall and winter months, can be particularly serious in children under one year of age and in older adults or people with other comorbidities. Because of the precautions taken during the COVID-19 pandemic, recent RSV infections have deviated from the usual seasonal trend. The purpose of this study was to assess the overall morbidity and mortality in patients with RSV.

**Methods:**

We conducted a multicenter historical cohort study of adult patients hospitalized for laboratory-confirmed RSV-related diseases in Ascension hospitals in southeast Michigan between January 2017 and December 2021. Hospitalized patients were identified using ICD 10 codes for RSV-related diseases. Electronic medical records were reviewed. Data were analyzed using the chi squared test, Student’s t test, and the Mann-Whitney U test using SPSS v. 29.0.

**Results:**

Of 360 patients, the mean (sd) age of the cohort was 69.9 + 14.7 years, 228 (63.5%) were female and 227 (63%) were white. The mean body mass index (BMI) was 30.6 + 9.7 kg/m^2^ and 56.1% were smokers. The most common comorbidities were hypertension (66.7%), chronic lung diseases (57.2%), obesity (33.6%), diabetes mellitus (31.1%) and congestive heart failure (26.1%). The mean Charlson weighted index of comorbidity was 2.2 ± 1.9. Cough (79.2%) and shortness of breath (72.8%) was most common symptoms. Admission chest x-rays were abnormal in 44.7% of cases. RSV upper and lower respiratory tract infections (RTI) were present in 58.4% and 41.6% of patients, respectively. Antibiotics were given in 56.9% of patients. Mechanical ventilation was required in 11% and intensive care in 14.2 % of patients. Other complications were acute kidney injury in 23.3%, encephalopathy in 11.4%, and liver injury in 3.6 %; 13 (3.6%) patients died and 21 (5.8%) were readmitted within 30 days to an Ascension hospital.

**Conclusion:**

Our study finds elderly patients hospitalized with RSV had substantial comorbidities. While overall mortality was low, the hospital course and complications suggest a disease that was costly both in human and financial suffering. Our study supports a need for RSV preventable initiatives and immunizations for this susceptible adult population.

**Disclosures:**

**All Authors**: No reported disclosures

